# Sialylation on vesicular integrin β1 determined endocytic entry of small extracellular vesicles into recipient cells

**DOI:** 10.1186/s11658-024-00562-0

**Published:** 2024-04-01

**Authors:** Meixuan Lin, Xiaoqiang Xu, Xiaoman Zhou, Hui Feng, Ruili Wang, Yunyun Yang, Jing Li, Ning Fan, Yazhuo Jiang, Xiang Li, Feng Guan, Zengqi Tan

**Affiliations:** 1 Key Laboratory of Resource Biology and Biotechnology in Western China, Ministry of Education, Provincial Key Laboratory of Biotechnology, College of Life Sciences, Northwest University, Xi’an, China; 2 Department of Urology, Provincial People’s Hospital, Xi’an, China; 3 Institute of Hematology, School of Medicine, Northwest University, Xi’an, China

**Keywords:** Sialic acid, Small extracellular vesicles, Integrin β1, Bladder cancer, Matrix fibronectin

## Abstract

**Background:**

Small extracellular vesicles (sEV) are closely associated with the development and metastasis of many types of mammalian cancer. Glycoconjugates are highly expressed on sEV and play important roles in sEV biogenesis and their interaction with other cells. However, the study on vesicular glycoconjugates are far behind proteins and nucleic acids. Especially, the functions of sialic acids which are the terminal components of glycoconjugates, are poorly understood in sEV.

**Methods:**

Sialic acid levels on sEV from plasma and bladder cancer cells were determined by ELISA and lectin blotting. Effects of sialylation on sEV uptake were determined by flow cytometry. Vesicular glycoproteins bearing sialic acids responsible for sEV uptake was identified by proteomics and density gradient centrifugation, and their site-specific sialylation functions were assayed by *N*-glycosylation site mutation. Effects of integrin β1 bearing sialic acids on the pro-metastatic function of sEV in vivo were explored using Balb/c nu/nu mice.

**Results:**

(1) Increased sialic acid levels were observed in sEV from malignant bladder cancer cells. (2) Elimination of sialic acids on sEV impaired sEV uptake by recipient cells. (3) Vesicular integrin β1 bearing sialic acids was identified to play a key role in sEV uptake. (4) Desialylation of the hybrid domain of vesicular integrin β1 inhibited its binding to matrix fibronectin, and reduced sEV entry into recipient cells. (5) Sialylation on integrin β1 affected pro-metastatic function of sEV in Balb/c nu/nu mice.

**Conclusions:**

Taken together, our findings indicate important functional roles of sialic acids in sEV uptake and reprogramming plasticity of surrounding normal epithelial cells.

**Graphical Abstract:**

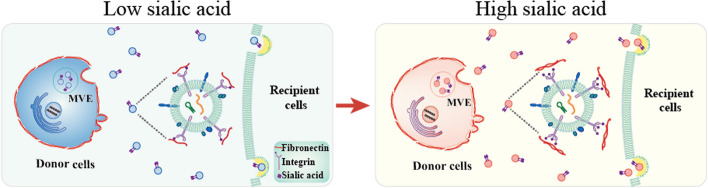

**Supplementary Information:**

The online version contains supplementary material available at 10.1186/s11658-024-00562-0.

## Background

Sialic acids, constitute the terminal group on carbohydrate chains of glycoproteins and glycolipids. Two major sialic acids, *N*-acetylneuraminic acid (Neu5Ac) and *N*-glycolylneuraminic acid (Neu5Gc) are widely distributed in animal tissues. The main form of sialic acids in human tissues is Neu5Ac. Hypersialylation (elevated sialic acid level) is frequently present on tumor cell surfaces [1], which can interfere with immune activation, thereby protecting tumor cells from recognition and attack by the immune system [2, 3]. Hypersialylation also enhances tumor proliferation and facilitates tumor angiogenesis and metastasis [4]. Notably, L.K. Mahal’s group reported the presence of sialic acids on exosomes, a type of small extracellular vesicles (sEV) [5].

sEV are nano-sized, spherical, bilayer membrane vesicles released by cells into the extracellular microenvironment under both normal and pathological conditions [6]. Tumor cell-derived sEV package various bioactive ingredients (including proteins, messenger RNAs, and microRNAs) and deliver such cargoes to recipient cells [7–9], and therefore play key roles in angiogenesis [10], tumor metastasis [11], and tumor microenvironment remodeling [12, 13].

Surfaces of sEV, like those of cell membranes, are covered by large amounts of glycoconjugates, including glycosphingolipids, proteoglycans, and glycoproteins [14]. Specific vesicular glycoconjugates are potential novel markers for the cancer screening and early diagnosis, and play essential roles in sEV biogenesis, uptake of sEV, and microenvironment reprogramming. For example, distinctive glycosylation compositions and patterns (“glycosignatures”) on vesicular proteins may serve as sorting motifs [15]. B cell-derived sEV with high α2,3-linked sialic acid levels promoted their binding to CD169^+^ macrophages [16]. In mouse melanoma models, *N*-glycosylation regulated sEV biogenesis, and metabolic inhibition of the *N*-glycosylation maturation suppressed sEV secretion [17]. The pro-metastatic function of sEV was dependent on a low level of bisecting GlcNAc modification in breast cancer cells [18]. However, in comparison with packaged proteins and nucleic acids, functions of vesicular glycoconjugates (particularly sialic acids on sEV) are poorly understood.

In this study, we investigated sialylation levels on sEV derived from the plasma from bladder cancer patients and cultured bladder cancer cells, and explored the capability of sialylation on sEV to modulate their uptake by recipient cells.

## Methods

### Cell lines and cell culture

Transitional carcinoma cell lines T24 was from the Cell Bank of the Chinese Academy of Sciences (Shanghai, Cat: SCSP-536). Human normal bladder mucosal epithelial HCV29, benigh non-muscle-invasive bladder cancer KK47, and highly malignant invasive bladder cancer YTS-1 cell lines [19–21], were kindly gifted by Dr. Sen-itiroh Hakomori (The Biomembrane Institute; Seattle, WA, USA). All cell lines were cultured in RPMI 1640 medium (HyClone; Provo, UT, USA) with 10% fetal bovine serum (FBS) (Biological Industries; Beit Haemek, Israel), 100 IU/mL penicillin, and 100 μg/mL streptomycin at 37 °C in 5% CO_2_ atmosphere.

### Generation of stable transfectants

Human full-length and site-directed mutant integrin β1 were constructed in our laboratory [22]. Full-length and mutant integrin β1 with flag tag were cloned into the pLVX-AcGFP-N1 plasmid (Takara; Shiga, Japan), and transfected into YTS-1 cells as described previously [18]. Stable transfectants were selected using puromycin and confirmed by western blotting.

Chemically synthesized oligonucleotides encoding integrin β1 short hairpin RNA (shRNA) were inserted in lentiviral plasmid Tet-pLKO-puro (Addgene plasmid #21915), and transfected into YTS-1 (termed Y-shβ1).

### Western blotting analysis

Total proteins were isolated from cells with RIPA buffer (1% Triton X-100, 5% glycerol, 0.5% sodium deoxycholate, 50 mM Tris, pH 7.2, 0.1% SDS, 150 mM NaCl, 10 mM MgCl_2_) containing 1% protease inhibitor and phosphatase inhibitor. The lysate was centrifuged at 14,000×*g* for 15 min at 4 °C. The supernatant was collected, and protein concentration was quantified by bicinchoninic acid (BCA) assay (Beyotime Biotechnology; Jiangsu, China). Proteins were separated by SDS-PAGE and transferred onto PVDF membranes (Bio-Rad; Hercules, CA, USA). Membranes were blocked with 5% (w/v) bovine serum albumin (BSA) in Tris-buffered saline with Tween-20 (TBST) for 1 h at 37 °C, probed with primary antibodies overnight at 4 °C, and incubated with appropriate HRP-conjugated secondary antibody. Bands were visualized by enhanced chemiluminescence (ECL) (Vazyme Biotech; Nanjing, China).

Primary antibodies involved in this study: TSG101 (ab83), Neu1 (ab197020), Integrin β1 (ab183666), CD63 (ab134045; Abcam; Cambridge, UK); Calnexin (2679), β-Tubulin (2146S), Alix (2171S; Cell Signaling Technology; Danvers, Massachusetts, USA); GAPDH (60004-1-lg; Proteintech Group; Inc Rosemont, USA); Fibronectin (sc271098; Santa Cruz Biotechnology; USA); Flag (M20008L; PharmaTech; Shanghai, China). All Primary antibodies were used at a ratio of 1:1000 (v/v).

### Lectin blotting analysis

Proteins were separated by SDS-PAGE as described above. PVDF membranes were blocked with phosphate-buffered saline with Tween-20 (PBST) containing 3% (w/v) BSA, incubated with SNA (B-1305) or MAL-II (B-B-1265, Vector Laboratories; Newark, CA, USA) for 12 h at 4 °C, washed with PBST, and incubated with HRP labeled streptavidin (ABC reagent, VECTASTAIN ABC Kit; Vector Laboratories). Bands were visualized by ECL. All lectins are used at a ratio of 1:1000 (v/v).

### Immunoprecipitation (IP)

Total proteins (1 mg) were incubated with 1 μg primary antibody for 2 h at 4 °C, then added with 20 μL Protein A/G Plus-Agarose. The mixture was incubated overnight at 4 °C with rotation, washed with PBS, denatured with loading buffer for 10 min at 100 °C, and analyzed by western blotting. Antibodies used in IP assay: Integrin β1 (ab183666); Flag (M20008L).

### Preparation of conditioned medium (CM)

Cells were incubated in the FBS-free medium for 48 h. The supernatant was collected and centrifuged at 500×*g* for 10 min, and then filtered with 0.22 μm filter as CM.

### sEV purification by differential ultracentrifugation

To prepare sEV, CM was sequentially centrifuged at 2000×*g* for 20 min, 10,000×*g* for 30 min at 4 °C, and ultracentrifuged twice at 100,000×*g* (model Optima XE-100; Beckman Coulter Life Sciences; Indianapolis, IN, USA) for 70 min. Pellets were resuspended in PBS and stored at − 80 °C.

### sEV purification by density gradient centrifugation

sEV were separated by density gradient centrifugation as described previously [23]. Briefly, 40%, 20%, 10%, and 5% (w/v) iodixanol solutions (OptiPrep; Axis-Shield PoC; Oslo, Norway) were prepared by diluting with 0.25 M sucrose/10 mM Tris, pH 7.5, placed in 14 × 89 mm Ultra-Clear tubes, and ultracentrifuged at 100,000×*g* for 18 h. Twelve fractions were collected, pelleted by ultracentrifugation (100,000×*g* for 3 h), resuspended in PBS, loaded on SDS-PAGE, and analyzed by western blotting.

### Transmission electron microscopy (TEM) and nanoparticle tracking analysis (NTA)

Purified sEV were morphologically characterized by TEM (model H-7650; Hitachi; Tokyo) at 80 kV as described previously [18]. sEV size distribution was evaluated by NTA (model NanoSight LM10; Malvern Instruments; Malvern, UK).

### Removal of sialic acids

Cells in 12-well plates or sEV were treated with 0.05 μg/μL sialidase (S10170, Yuanye Bio-Technology Co.; Shanghai, China) for 1.5 h at 37 °C, which could catalyze the release of the α2,3- and α2,6-sialic acids from glycoprotein. The desialylated sEV were confirmed by lectin blotting and used for further uptake assay.

### Identification of sEV proteins

sEV proteins (50 μg) were added in a size-exclusion spin ultrafiltration unit (10 kD; Millipore, Marlborough, MA, USA), denatured with urea, reduced with dithiothreitol (DTT), alkylated with iodoacetamide (IAM), digested with trypsin, desalted with C18 reversed-phase column, lyophilized, and analyzed by LTQ Orbitrap mass spectrometry (MS) (Thermo Fisher Scientific; San Jose, CA, USA) as described previously [24].

The proteomic data have been deposited to the ProteomeXchange Consortium via the PRIDE, partner repository with the PXD036973 and 10.6019/PXD036973.

### sEV uptake

50 μg sEV were labeled with ExoTracker as described previously [25]. In brief, sEV were incubated with ExoTracker for 1 h at room temperature, and excess ExoTracker were removed using 10 kD ultrafiltration unit. Cells in 24 wells plates were incubated with labeled sEV for 1 h, detached with trypsin, rinsed with PBS, and analyzed by flow cytometry (ACEA Biosciences; San Diego, CA, USA).

### ExoTracker labeling efficiency of sEV

50 μg sEV or sialidase-treated sEV were labeled with ExoTracker as described above, and incubated with 50 μL CD63 exosome capture beads (ab239686; Abcam; Cambridge, UK) in the dark overnight at room temperature. Fluorescence signal of sEV were analyzed by flow cytometry.

### sEV biotin labeling and uptake detection

sEV were extracted and resuspended to 0.5 μg/μL with PBS buffer, and incubated with 10 μmol/L NHS-LC-biotin (Sigma-Aldrich) with shaking for 15 min at room temperature. Excess NHS-LC-biotin were removed using 10 kD ultrafiltration unit, and the protein concentration was determined. Cells were incubated with 50 μg of labeled sEV for 2 h or 24 h. The amount of sEV entering the cells was detected by western blotting. Recipient cells treated with non-labeled sEV and sialidase were used as control group.

### sEV integrin β1 blocking

sEV were incubated with anti-integrin β1 neutralizing antibodies (102201, BioLegend Inc., San Diego, CA, USA) or mouse IgG (A7028, Beyotime Biotechnology; Jiangsu, China) at 1:50 (w/w) for 1 h on the ice.

### Patient samples

Plasma samples from bladder cancer patients and healthy volunteers were obtained from Shaanxi Provincial People’s Hospital. Written informed consent was obtained from all patients, in accordance with the Declaration of Helsinki guidelines. Experiments using human plasma were approved by the Research Ethics Committee of Northwest University. Characteristics of bladder cancer patients and healthy volunteers were shown in Additional file 1: Tables S1 and S2.

### Enzyme-linked immunosorbent assay (ELISA) of sialic acids on sEV

To determine levels of sialic acid on sEV, sialic acid on CD63 or sialic acid on integrin β1, plasma samples or plasma lysates (plasma lysed with RIPA buffer) were respectively added onto multi-well ELISA plates pre-coated with anti-CD63 antibody or anti-integrin β1 antibody, and incubated for 12 h at 4 °C. The plates were washed with PBST, blocked with BSA, and incubated with biotinylated SNA/ MAL-II for 30 min at 37 °C. The plates were washed with PBST, and incubated with HRP labeled streptavidin for 30 min at 37 °C. The mixture was incubated with 3,3′,5,5′-tetramethylbenzidine (TMB) reagent (Promega; Madison, USA), and quenched with 2 M sulfuric acid. The absorbance at 450 nm was measured.

To determine sialic acid levels, total CD63 levels or total integrin β1 levels in plasma, plasma samples or plasma lysates (plasma lysed with RIPA buffer) were respectively added onto ELISA plates, and incubated for 12 h at 4 °C. The plates were blocked, probed with lectins, antibody against CD63 or antibody against integrin β1, and visualized with TMB reagent as described above.

To determine levels of sialylated integrin β1 on sEV from plasma samples, sEV separated from plasma by ultra-centrifugation was added onto multi-well ELISA plates pre-coated anti-integrin β1 antibody, blocked with BSA, probed with biotinylated SNA/MAL-II, and visualized with TMB reagent as described above.

### Cell apoptosis assay

Cells were incubated with sEV (final concentration of 100 μg/mL) for 48 h at 37 °C, detached with trypsin, centrifuged at 1000×*g* for 5 min, washed with PBS, resuspended in 100 μL 1× binding buffer containing 2.5 μL APC-Annexin V and 2.5 μL 7-AAD (BioLegend; San Diego, CA, USA), incubated for 20 min in the dark, and analyzed by flow cytometry.

### Cell proliferation assay

Cells with a confluence of 40%-50% were incubated with CM or sEV (final concentration of 100 μg/mL) for 24 h, processed for EdU incorporation using baseclick EdU Kit (Promega; Madison, USA), and analyzed by flow cytometry.

### Transwell assay

Cell culture inserts (pore size 8 μm; Corning; Corning, NY, USA) were used as per the manufacturer’s instructions. Cells (1 × 10^4^) were starved in serum-free medium added sEV (final concentration of 100 μg/mL) for 24 h, seeded in upper chambers, added with RPMI 1640 complete medium to the bottom chamber, and incubated for 36 h. Cells on the upper surface of each filter were removed with cotton swabs, which migrated across the membrane were stained with 0.1% crystal violet, and photographed under microscopy.

### sEV pre-conditioning of mice

All mouse experiments were approved by the Animal Care and Use Committee of Northwest University. sEV (20 μg in 100 μL PBS) were centrifuged at 4600×*g* for 1 min at 4 °C to remove sedimentable aggregates, then i.v. injected into 6- to 8-week-old Balb/c nu/nu mice twice per week.

### Liver/lung colonization studies

Six- to 8-week-old female Balb/c nu/nu mice pre-conditioned with sEV were injected with 2 × 10^6^ YTS-1 cells via tail vein. Mice were euthanized 8 weeks after injection. Lungs were fixed, sectioned, and stained with haematoxylin and eosin (H&E).

### Data analysis

Each experiment was performed with three or more replicates. Statistical analyses were performed using the software program GraphPad Prism V. 8.0. Data from two groups were compared by two-tailed Student’s *t*-test, and results were presented as mean ± SD. Differences with *p* < 0.05 were considered statistically significant. Notations in figures: *, *p* < 0.05; **, *p* < 0.01; ***, *p* < 0.001.

## Results

### Enriched sialylation on sEV from bladder cancer cells and plasma

**Fig. 1:**
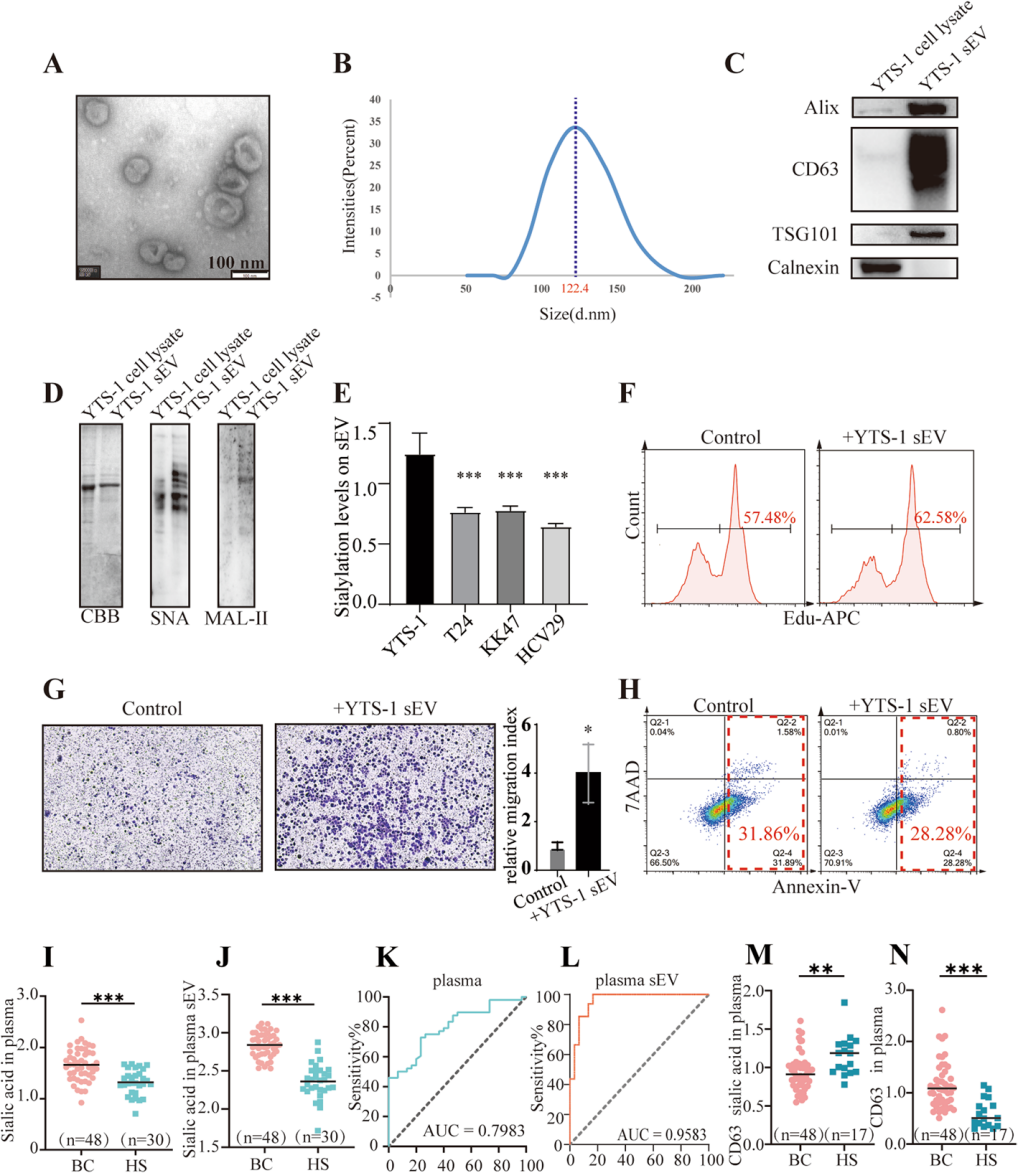
Enriched sialylation on sEV of bladder cancer cells and serums. **A** YTS-1 sEV morphology (TEM image). **B** The particle size of YTS-1 sEV, determined by nanoparticle tracking analysis (NTA). **C** Expression of sEV markers, analyzed by western blotting. **D** Sialic acid levels in YTS-1 cells lysate and YTS-1 sEV. Coomassie Brilliant Blue (CBB). **E** Sialic acid levels on sEV from YTS-1, T24, KK47 and HCV29 cells by ELISA. **F**–**H** Proliferation (**F**), migratory ability (**G**), apoptosis (**H**) of HCV29 treated with YTS-1 sEV. **I** Sialylation of plasma from bladder cancer patients and healthy subjects by ELISA. BC: bladder cancer patients. HS: healthy subjects. **J** Sialylation of plasma sEV by ELISA. **K** Bladder cancer ROC curve based on plasma sialylation. AUC: area under the curve. **L** Bladder cancer ROC curve based on plasma sEV sialylation. **M** Levels of sialylated CD63 in plasma detected by ELISA. **N** Levels of total CD63 in plasma detected by ELISA

CM from bladder cancer YTS-1 and KK47 promoted the proliferation and migration, and inhibited the apoptosis of normal bladder epithelial HCV29 (Additional file 1: Figures S1–3). Knockdown of Rab27A (Additional file 1: Figure S4A, B), a key gene involved in sEV secretion [26], reduced sEV secretion level (Additional file 1: Figure S4C) and suppressed pro-malignant functions of CM (Additional file 1: Figures S5–7), indicating an important role of sEV from CM in affecting the cellular behaviors of recipient cells. Thus, we isolated sEV from bladder cancer cells by differential centrifugation, and they displayed sphere-like morphology (Fig. 1A), diameter ~ 100 nm (Fig. 1B), high expression of CD63, Alix, and TSG101, and low expression of the ER-associated chaperone protein calnexin (Fig. 1C). In view of the dysregulation of sialic acid levels in bladder cancer in our previous study [27], and the essential functions of sialic acids in a variety of physiological and pathological processes [4], lectin blotting of sEV was performed, revealing that α2,3- and α2,6- sialic acids, recognized by lectins MAL-II and SNA respectively, were present on the YTS-1 derived sEV (Fig. 1D). sEV from YTS-1 displayed higher sialylation levels than which from other bladder cancer cells T24 and KK47, and normal epithelial cells HCV29 (Fig. 1E). Thus, sEV from YTS-1 with high sialylation levels were selected for further study. sEV from YTS-1 could be taken up by HCV29 (Additional file 1: Figure S8), resulting in an increased proliferation (Fig. 1F), enhanced migration (Fig. 1G), and decreased apoptosis (Fig. 1H).

Sialic acid levels of both plasma and plasma sEV were significantly higher in bladder cancer patients than which in healthy subjects (Fig. 1I, J), and the sialic acid level in plasma sEV (vs. plasma per se) showed a stronger association with bladder cancer status (Fig. 1K, L). Sialic acid levels on CD63 were significantly lower in plasma from bladder cancer patients, accompanied by an elevated total CD63 level (Fig. 1M, N), suggesting that changes in sialic acids on sEV are not stem from the elevated sialylated CD63 in bladder cancer. These data indicate the high sialylation on sEV from bladder cancer cells and patient plasma.

### Effects of sialylation on sEV entry into recipient cells

**Fig. 2:**
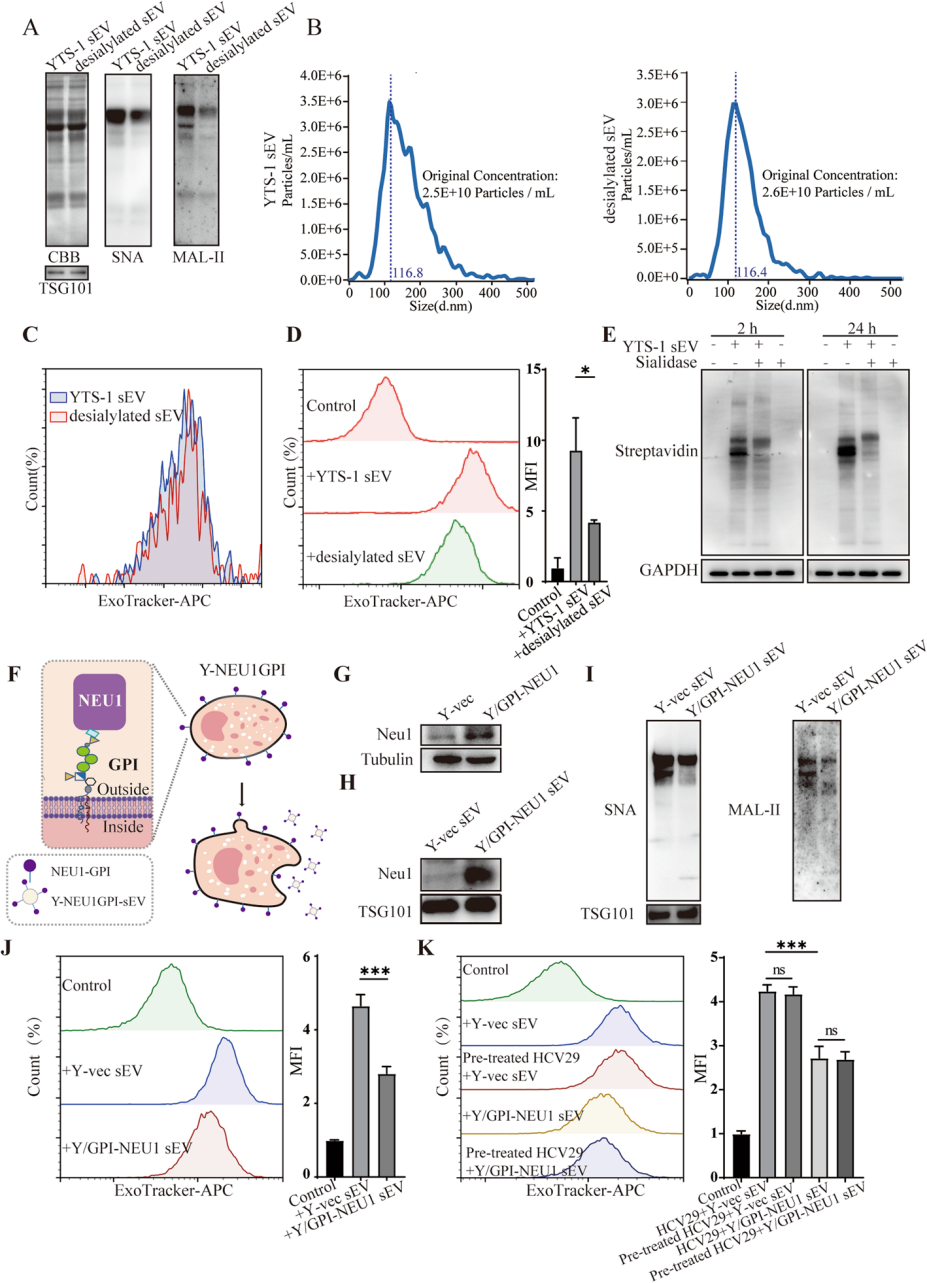
Effects of sialylation on sEV entry into recipient cells. **A** Sialic acid levels on sialidase-treated sEV. CBB: Coomassie Brilliant Blue staining. **B** The particle size and particle count of YTS-1 sEV and desialylated sEV determined by nanoparticle tracking analysis (NTA). **C** The fluorescence signal of sialylated and desialylated sEV labeled by ExoTracker determined by flow cytometry. **D** Effect of sialylation on sEV uptake, analyzed by flow cytometry. **E** Levels of biotin-labeled vesicular proteins in recipient cells. **F** Y/GPI-NEU1 cell construct (schematic). **G** The expression of GPI anchored NEU1 in Y/GPI-NEU1 cells, analyzed by western blotting. Tubulin was used as loading control. **H** The expression of GPI anchored NEU1 in Y/GPI-NEU1 sEV. TSG101 was used as loading control. **I** Sialic acid levels in Y/GPI-NEU1 sEV. TSG101 was used as loading control. **J** Uptake of Y/GPI-NEU1 sEV and Y-vec sEV by HCV29. **K** Uptake of Y/GPI-NEU1 sEV and Y-vec sEV by HCV29 and sialidase pre-treated HCV29

To study the biological role of sialylation on sEV, sEV were de-sialylated with the sialidase (Fig. 2A). Equal amounts of YTS-1 sEV and desialylated sEV (Fig. 2B) were labeled with ExoTracker with the equal labeling efficiency (Fig. 2C), and their uptake was determined. Compared to sialidase-treated YTS-1 sEV (termed desialylated sEV), YTS-1 sEV entry into recipient cells was higher, determined by flow cytometric analysis (Fig. 2D). Furthermore, YTS-1 sEV and desialylated sEV were labeled with NHS-biotin, and these labeled sEV were both internalized by recipient cells (Fig. 2E). Consistently, the uptake of YTS-1 sEV by HCV29 was significantly higher than that of desialylated sEV (Fig. 2E).

In addition, the human sialidase NEU1 was overexpressed and anchored to the YTS-1 membrane via glycosylphosphatidylinositol (GPI) (Fig. 2F, G). NEU1 expression was significantly higher in sEV from NEU1 overexpressed cells (termed Y/GPI-NEU1), relative to Y-vec sEV (Fig. 2H), accompanied by lower sialic acid levels in Y/GPI-NEU1 sEV (Fig. 2I). The uptake of Y/GPI-NEU1 sEV by recipient cells was much lower than that of Y-vec sEV (Fig. 2J).

To rule out the effect of recipient cell sialylation on sEV uptake, HCV29 was treated with sialidase, resulting in no significant change in sEV uptake (Fig. 2K). These findings indicate that sialylation on sEV, other than sialylation on recipient cells, affect the uptake of sEV by recipient cells.

### Identification of sialylated integrin β1 on sEV

**Fig. 3:**
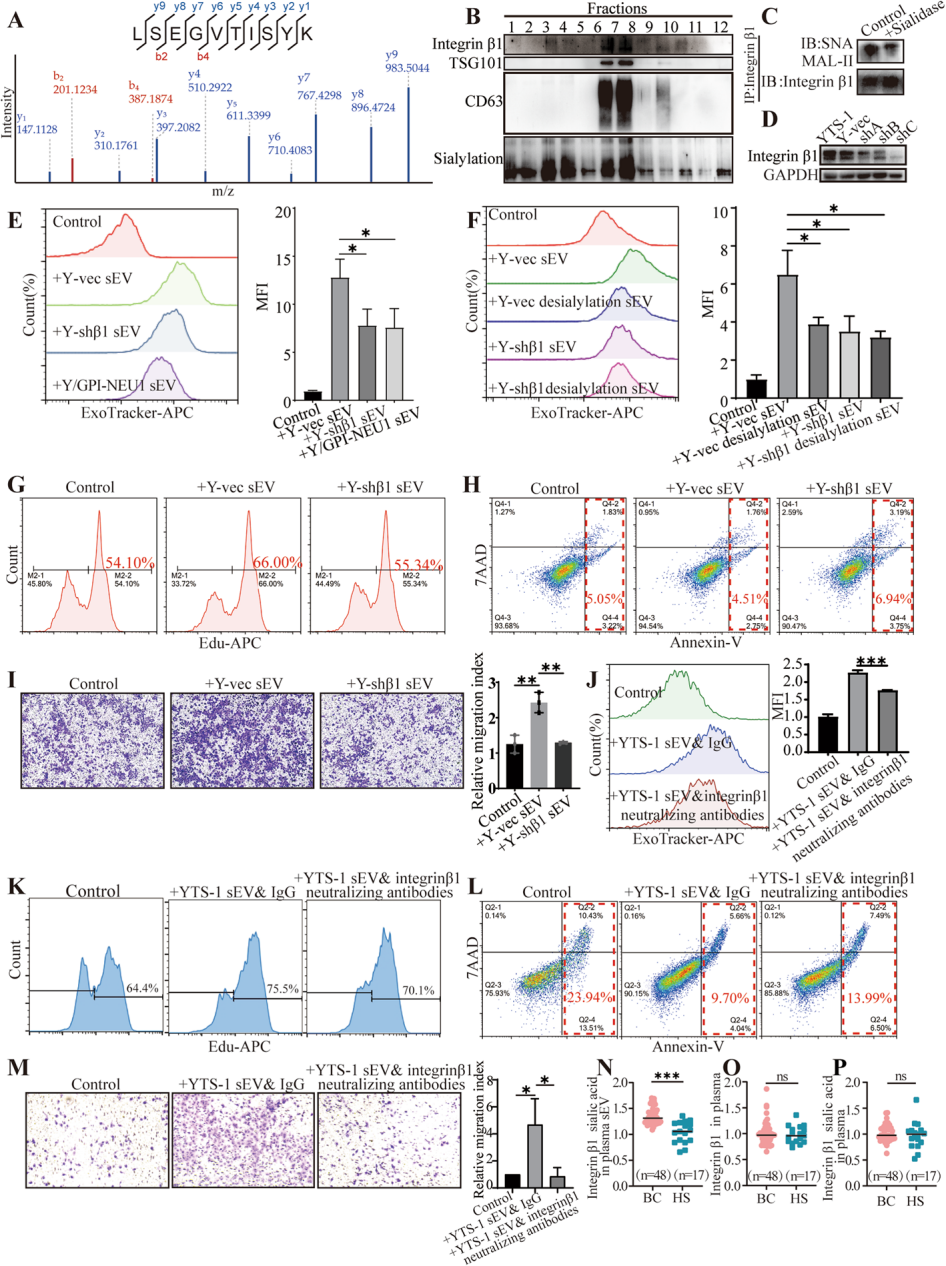
Identification of sialylated integrin β1 on sEV. **A** Identification of integrin β1 on YTS-1 sEV by MS. **B** YTS-1 sEV were purified by density gradient centrifugation. Levels of two sEV markers (TSG101, CD63) and integrin β1 in fractions 1–12 were analyzed by western blotting, and sialic acid levels were analyzed by lectin blotting. **C** Integrin β1 on sEV was enriched by IP and sialylation of vesicular integrin β1 was analyzed by lectin blotting. **D** Integrin β1 knockdown in YTS-1 (termed shA/B/C). shC (termed Y-shβ1) was used for further assay. GAPDH was used as loading control. **E** Uptake of ExoTracker-labeled sEV from Y-vec, Y-shβ1, and Y/GPI-NEU1. **F** Y-vec sEV and Y-shβ1 sEV were treated with/ without sialidase and ExoTracker-labeled, and their uptake by HCV29 was analyzed by flow cytometry. **G**–**I** HCV29 was treated with Y-vec sEV or Y-shβ1 sEV, and proliferation (**G**), apoptosis (**H**), and migratory ability (**I**) were determined. **J** Effects of integrin β1 blockage on sEV uptake. **K**–**M** HCV29 was incubated with YTS-1 sEV pre-treated with IgG or integrinβ1 neutralizing antibodies, and proliferation (**K**), apoptosis (**L**), and migratory ability (**M**) were determined. **N** Levels of sialylated integrin β1 on sEV from plasma, determined by ELISA. **O** Levels of total integrin β1 in plasma determined by ELISA. **P** Levels of sialylated integrin β1 in plasma determined by ELISA

sEV influence the behaviors of recipient cells through the transfer of bioactive ingredients, such as DNA, RNA, and proteins [6, 28]. To identify potential sialylated vesicular proteins responsible for sEV uptake, we performed the proteomic analysis of YTS-1 sEV, revealing the presence of integrin β1 in YTS-1 sEV (Fig. 3A). Moreover, our findings confirmed that integrin β1 was predominantly localized within components 6 and 7 of the density gradient fractions, aligning with the positions of sEV markers TSG101 and CD63. Notably, sialic acid bands were evident in components 6 and 7, indicating the coexistence of vesicular integrin β1 and sialylation (Fig. 3B). The sialylation on integrin β1 was further validated through IP and lectin blotting. Treatment with sialidase resulted in a significant reduction in sialylation on vesicular integrin β1 (Fig. 3C). These results offer compelling evidence for the presence of sialic acid modification on vesicular integrin β1.

To assess the functional role of vesicular integrin β1, integrin β1 was silenced in YTS-1 cells (termed Y-shβ1) (Fig. 3D). Integrin β1 knockdown have no effects on sEV distribution, but changed sialic acid expression patterns in sEV fractions (Additional file 1: Figure S9). The uptake of Y-shβ1 sEV and Y/GPI-NEU1 sEV by recipient cells were notably diminished by either integrin β1 knockdown or sEV desialylation (Fig. 3E). Notably, the combination of these two treatments did not lead to a further reduction in sEV uptake (Fig. 3F). Collectively, these observations underscore the pivotal role of sialic acids on integrin β1, rather than sialic acids or integrin β1 independently, in facilitating sEV uptake.

Proliferation of recipient cells was enhanced strongly by sEV from vector control YTS-1 cells (termed Y-vec sEV), and slightly by Y-shβ1 sEV (Fig. 3G). Apoptosis was reduced by Y-vec sEV, and increased by Y-shβ1 sEV (Fig. 3H). Migratory ability was enhanced by Y-vec sEV, but unaffected by Y-shβ1 sEV (Fig. 3I). The absence of shRNA targeting integrin β1 in sEV (Additional file 1: Figure S10) excluded the direct interference of shRNA with recipient cells. Similar results were observed in recipient cells treated with CM from control cells or which from Y-shβ1 cells (Additional file 1: Figures S11–13). Moreover, the neutralizing antibody against integrin β1 was employed to validate the biological role of vesicular integrin β1, showing that sEV uptake (Fig. 3J), proliferation (Fig. 3K) and metastasis (Fig. 3M) of recipient cells were suppressed by the blockage of integrin β1, accompanied by the elevated apoptosis (Fig. 3L).

Levels of sialic acid on vesicular integrin β1 were significantly higher in plasma from bladder cancer patients (Fig. 3N), with no significant changes in both total integrin β1 and sialylated integrin β1 levels between plasma from bladder cancer patients and healthy donors (Fig. 3O, P), suggesting an elevated level of sialic acid on vesicular integrin β1, but not overall integrin β1, in plasma from bladder cancer patients.

### Effects of integrin β1 sialylation on sEV uptake

**Fig. 4:**
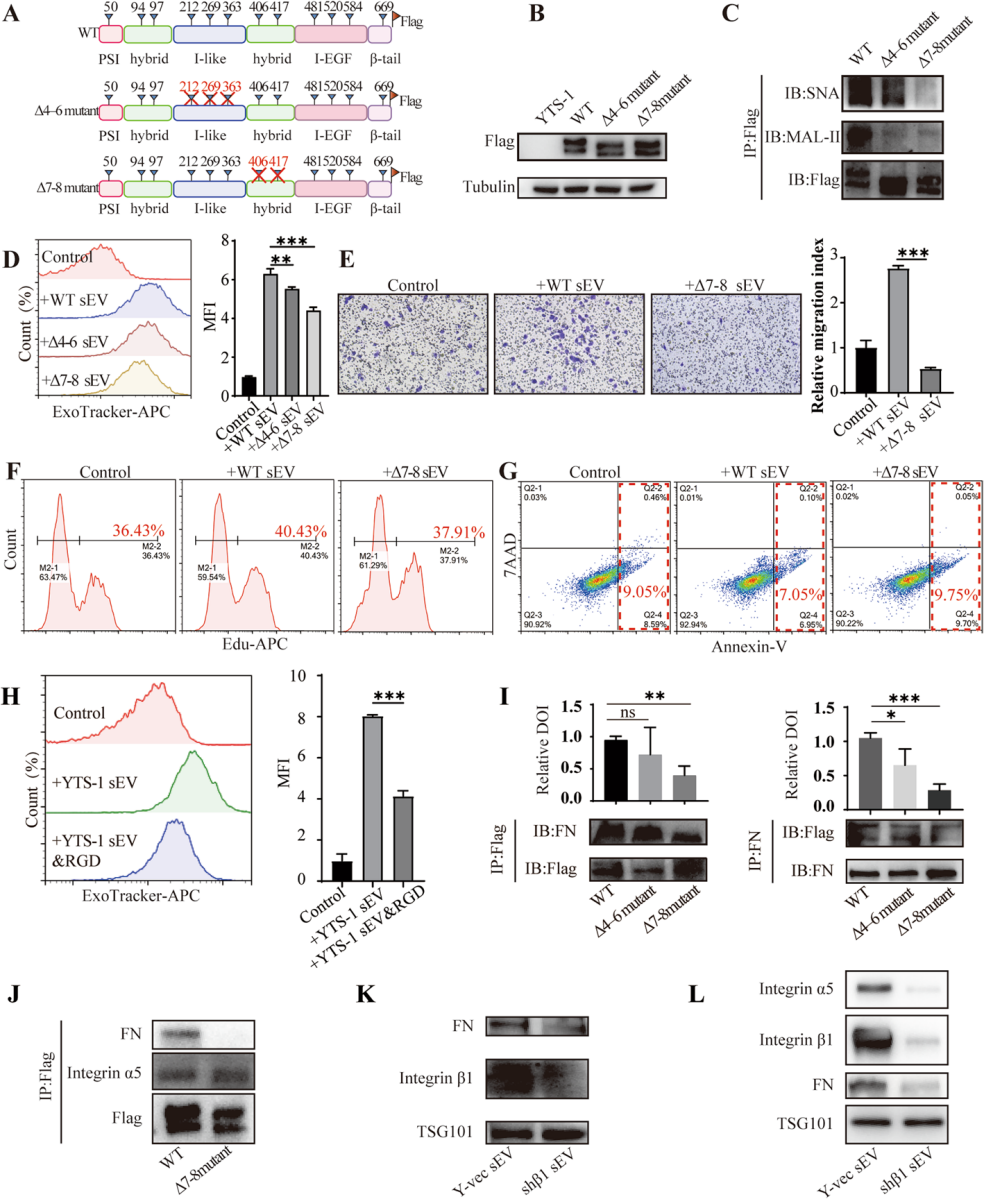
Effects of integrin β1 sialylation on sEV uptake. **A** Potential *N*-glycosylation sites (indicated by triangles) on integrin β1. Combined mutation to Asp of *N*-glycosylation sites 4–6 and 7–8 gave rise to Δ4–6 and Δ7–8. **B** Expression of WT integrin β1, Δ4–6, and Δ7–8 mutants. Tubulin was used as loading control. **C** Sialylation levels on integrin β1 in mutants by co-IP and western blotting. **D** Effects of mutant integrin β1 on sEV uptake. **E**–**G** HCV29 was treated with sEV from Y-vec, WT, and Δ7–8 mutant. Migratory ability (**E**), proliferation (**F**) and apoptosis (**G**) were determined. **H** Uptake of YTS-1 sEV in the presence of cilengitide (“RGD”). **I** Integrin β1/ FN interaction, assayed by co-IP and western blotting. **J** The interaction between integrin α5β1 and FN in cells expressing wild type integrin β1 and Δ7–8 mutant, assayed by co-IP and western blotting. **K** Integrin β1 and FN expression in sEV from Y-vec and Y-shβ1. TSG101 was used as loading control. **L** Integrin β1, α5 and FN levels in sEV from Y-vec and Y-shβ1. TSG101 was used as loading control

Integrin β1 is a typical glycoprotein characterized by its distinct structural domains, including a plexin-semaphorin-integrin (PSI) domain, an integrin-epidermal growth factor (I-EGF) domain, an I-like domain, both upstream and downstream of the hybrid domain, and a β-tail domain [29]. We further explored which domain of integrin β1 mainly bearing sialic acids using our previous established breast cancer cells expressing integrin β1 with mutant *N*-glycosylation sites in distinct domains (Additional file 1: Figure S14) [22]. Lectin blotting analysis unveiled a markedly lower α2,6-linked sialic acid level in hybrid domain mutant (termed Δ7–8 mutant) compared to which in the other three mutants (Additional file 1: Figure S15). To gain deeper insights into whether site-specific sialylation of integrin β1 could influence the endocytosis of sEV in bladder cancer cells, we overexpressed flag-tagged wild type (WT) integrin β1, I-like domain mutant (termed Δ4-6 mutant), and hybrid domain mutant (Δ7–8 mutant) in YTS-1 (Fig. 4A, B). IP assay revealed a notable reduction in sialylation levels, particularly α2,6-linked sialic acid, in hybrid domain mutant in comparison to the other groups (Fig. 4C). These findings strongly suggest that the critical integrin β1 sialylation sites are located at Asn 406 and 417 of hybrid domain.

In terms of cellular uptake, HCV29 cells exhibited significantly diminished internalization of sEV derived from Δ7-8 mutant (termed Δ7–8 sEV), when compared to sEV derived from cells overexpressing WT integrin β1 (termed WT sEV) or Δ4–6 mutant (termed Δ4–6 sEV) (Fig. 4D). Migratory ability and cell proliferation of recipient cells was reduced by Δ7-8 sEV compared to WT sEV (Fig. 4E, F). Apoptosis of recipient cells was strongly elevated Δ7-8 sEV treatment in comparison to WT sEV (Fig. 4G). Similar results were obtained in recipient cells treated with CM from YTS-1 cells expressing Δ7–8 mutant (Additional file 1: Figures S16–18).

Integrins function as heterodimeric, transmembrane cell adhesion receptors for extracellular matrix (ECM) molecules, particularly FN. The presence of the cyclic Arg-Gly-Asp (RGD) pentapeptide cilengitide, an integrin inhibitor known to disrupt FN-integrin interactions [30–32], led to a notable reduction in the uptake of YTS-1 sEV by recipient cells (Fig. 4H). Furthermore, the interaction between FN and integrin β1 was notably impaired in YTS-1 cells expressing Δ7-8 mutant due to the mutation of glycosylation sites on the hybrid domain (Fig. 4I). Integrin α5, the commonly reported α subunit of integrin β1, was interacted with integrin β1 and FN, and the interaction between FN and integrin α5β1 was suppressed in Δ7–8 mutant (Fig. 4J).

Recent studies demonstrated that FN could be sorted into sEV depends on binding to integrins such as integrin α5β1 [23], consistently, integrin β1 knockdown reduced FN and integrin α5 levels in Y-shβ1 sEV (Fig. 4K, L). We propose that *N*-glycosylation sites (assumed to bear sialic acid) strengthens the direct or indirect interaction between integrin β1 and FN, facilitates the entry of sEV into recipient cells, and reprograms plasticity of normal epithelial cells.

### Sialylation on integrin β1 affected pro-metastatic effects of sEV in vivo

**Fig. 5:**
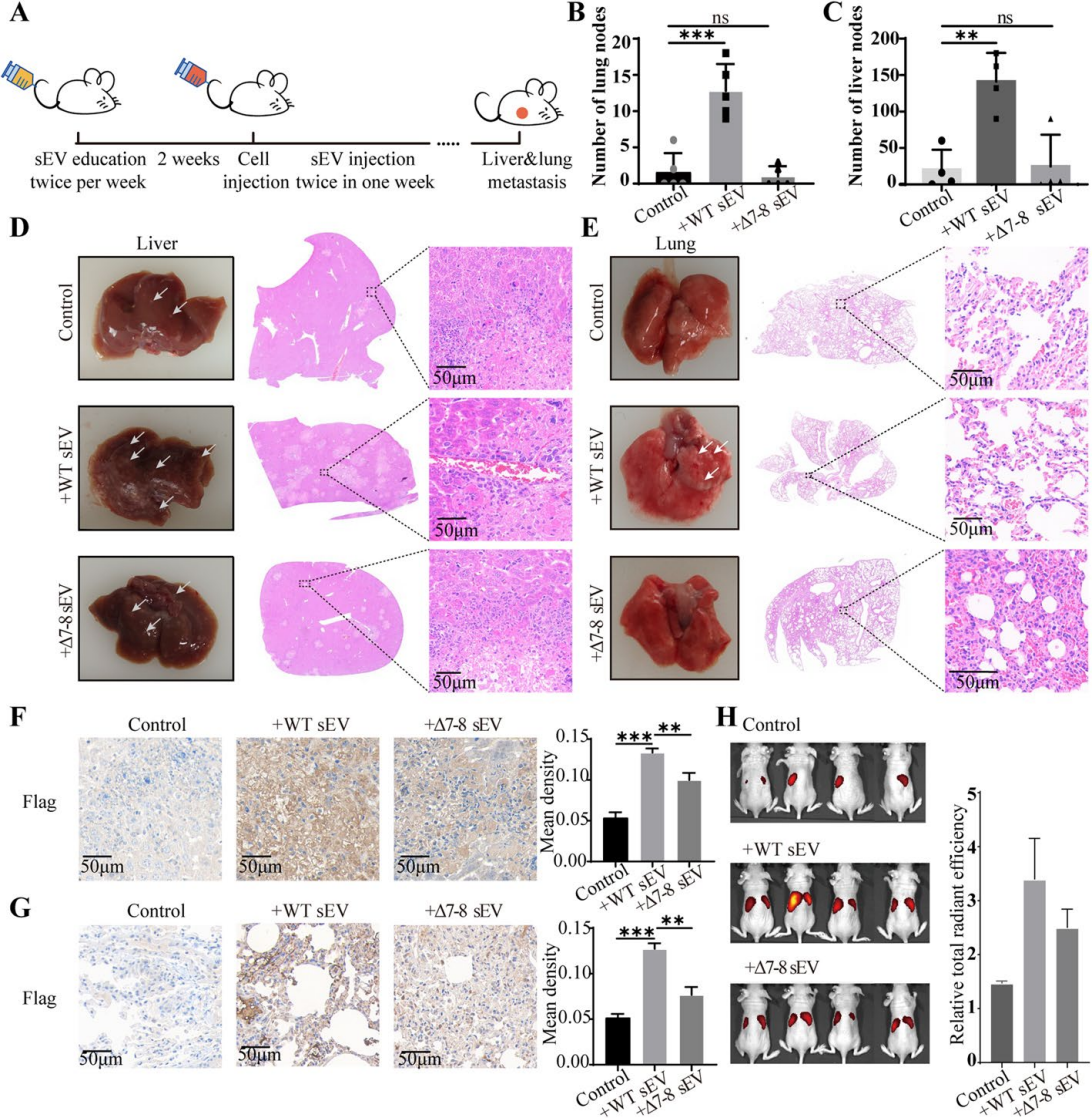
Effects of integrin β1 sialylation on the pro-metastatic function of sEV. **A** Experimental workflow (schematic). **B**–**E** Representative photographs and haematoxylin and eosin (H&E) staining of livers or lungs. **F**, **G** Flag-tagged WT or Δ7–8 mutant integrin β1 expression in liver (**F**) or lung (**G**) by immunohistochemistry. **H** Distribution of sEV in mice after injection of sEV by tail vein

It was reported that sEV were involved in the pre-metastatic niche formation, and vesicular integrins could determine organotropic metastasis [33]. Next, we examined whether *N*-glycosylation sites (assumed to bear sialic acid) on integrin β1 could affect the pro-metastatic function of sEV using the mouse model. The nude mice were preconditioned with WT sEV or Δ7-8 sEV, and then injected with YTS-1 cells (Fig. 5A). The incidence, numbers, and areas of liver /lung metastasis nodules were significantly enhanced by WT sEV, but not by Δ7–8 sEV (Fig. 5B–E). Immunohistochemistry analysis revealed that vesicular flag-tagged integrin β1 was significantly accumulated in the liver and lung of mice treated WT sEV, but not Δ7-8 sEV (Fig. 5F, G), revealing that the mutation of *N*-glycosylation sites (assumed to bear sialic acid) on integrin β1 decreased the residency of sEV in the liver and lung. Moreover, the half-life of sEV in mice was further evaluated. The WT sEV or Δ7-8 sEV were labeled with fluorescent dye CFSE and injected into the mice through the tail vein. After 24 h, it was found that more WT sEV was retained in the mice compared to Δ7-8 sEV (Fig. 5H), indicating that *N*-glycosylation (containing sialylation) on sEV could prolong the circulation time of sEV in vivo*.*

## Discussion

Glycoconjugates play crucial roles in various biological processes, encompassing cellular recognition, intercellular communication, protein folding, intracellular transport, and protein quality control [34, 35]. sEV bear an abundant array of glycoconjugates, much like the plasma membrane. The glycosignatures of sEV hold the potential as valuable resources for discovering new cancer biomarkers [36]. Sialic acids serve as terminal constituents of numerous glycoproteins and glycolipids, and are found to be presented on the sEV from breast cancer cells [15]. We observed elevated sialic acid levels in both plasma and plasma-derived sEV from individuals with bladder cancer. Notably, the sialic acid level on plasma-derived sEV emerged as a more effective discriminator for distinguishing between bladder cancer patients and healthy volunteers (Fig. 1K, L).

Furthermore, our investigation unveiled the critical role of sialylation on sEV in governing the process of sEV uptake. Specifically, we found that the internalization of sEV from bladder cancer cells into recipient cells was notably hindered through the actions of either desialylation or the knockdown of vesicular integrin β1. Sialic acids are pivotal contributors to cell adhesion and signal recognition due to their distinct properties. For example, sialylation acts as a shielding mechanism, concealing Gal or GalNAc residues and impeding recognition by the asialoglycoprotein receptor (ASGPR) [37]. Sialic acids serve as molecular pattern ligands recognized as “self” by sialic acid-binding immunoglobulin-type lectins (siglecs) [38]. Thus, sialylation on sEV might regulate the sEV uptake through the direct or indirect interaction between sialylated vesicular glycoproteins and receptor on recipient cells.

Sialylation modulates the activity of certain membrane proteins, particularly integrins. Sialylation of integrin β1 blocked cell adhesion to galectin-3 and inhibited galectin-3-induced apoptosis [39]. Hypersialylation of integrin β1 enhanced cell migration, and promoted colonic tumor progression by altering cell preference for certain types of ECM [40]. A 2022 study by O. Moscovitz’s group indicated that the terminal sialic acid on sEV is essential for their uptake by human monocytes [41]. By constructing different integrin β1 mutants, we observed a significant reduction in the overall sialic acid levels within the Δ7–8 mutant (Fig. 4C). Desialylation of the hybrid domain of vesicular integrin β1 significantly altered the uptake and pro-malignance of sEV (Fig. 4D–G).

Previous reports have highlighted that the positioning of FN on sEV is contingent upon its interaction with integrins [23], and the presence of FN on sEV facilitates their internalization by binding to membrane proteins on recipient cells [42]. In line with these findings, our observations align, as hindering the interaction between integrin β1 and FN through cilengitide treatment led to a comparable inhibition of sEV entry. Furthermore, the desialylation of hybrid domain of integrin β1 impaired the interaction between FN and integrin α5β1 (Fig. 4I, J), resulting in a reduced sEV uptake by recipient cells (Fig. 4D). In view of the present findings, we hypothesize that sialylation of vesicular integrin β1 facilitates its interaction with FN, and promotes endocytic entry of sEV into recipient cells.

Communication between cancer cells and the surrounding microenvironment is an essential process in cancer progression. Pancreatic cancer exosome DNAJB11 can promote pancreatic cancer development [43]. Tumor cell-derived sEV have the ability to promote tumor progression and survival by reprogramming or "educating" neighboring cells. For example, sEV derived from glioma cells under hypoxia promoted angiogenesis through phenotypic regulation of endothelial cells [44]. sEV released by breast cancer cells under hypoxia induced oncogenic changes in normal mammary epithelial cells [45]. sEV-mediated proliferation and migration of recipient human hepatocellular carcinoma cells were inhibited by the reduction of sialic acid level on sEV through ST6Gal-1 knockdown in donor cells [46]. In the present study, sialylated sEV (YTS-1 sEV), but not desialylated sEV, induced oncogenic properties (enhanced proliferation and migration; reduced apoptosis) in normal cells. We propose that the underlying mechanism also involves the enhancement of sialic acid level in recipient cells by uptake of heavily sialylated sEV, furthermore, sialylated vesicular integrin β1 might be recycled following endocytosis, leading to activation of responding signal pathways and oncogenic transformation of normal epithelial cells.

In another way, tumor-derived sEV could be uptaken by organ-specific cells and establish the pre-metastatic niche, while exosomal integrins determine the organotropic metastasis, e.g., exosomal integrin αvβ5 was associated with liver metastasis [33]. We found that reduced sialylation of integrin β1 resulted in reduced liver metastasis and lung metastasis of bladder cancer in vivo (Fig. 5D, E). It was suggested that sialylation affected the function of integrin, which in turn affected the domestication of the local and distant microenvironment by sEV.

## Conclusions

In conclusion, sialylated integrin β1 on sEV affects its entrance into recipient cells. Sialylation on sEV has may alter the biological function of sEV and has the potential to be a new indicator for bladder cancer diagnosis.

### Supplementary Information

**Additional file 1: Figure S1.** Proliferation of HCV29 treated with KK47 CM or YTS-1 CM. **Figure S2.** Apoptosis of HCV29 treated with KK47 CM or YTS-1 CM. **Figure S3.** Migratory ability of HCV29 treated with KK47 CM or YTS-1 CM. **Figure S4.** Knockdown of Rab27A in YTS-1. **Figure S5.** Migratory ability of HCV29 treated with Y-vec CM or Y-shRab27a CM. **Figure S6.** Proliferation of HCV29 treated with Y-vec CM or Y-shRab27a CM. **Figure S7.** Apoptosis of HCV29 treated with Y-vec CM or Y-shRab27a CM. **Figure S8.** Confocal microscopic imaging of sEV endocytosis. HCV29 were treated with fluorescence labeled YTS-1sEV. **Figure S9.** Levels of sialic acids, integrin β1, CD63 and TSG101 in sEV from integrin β1 silenced cells. **Figure S10.** shRNAs in sEV from Y-shβ1 cells by realtime PCR analysis. **Figure S11.** Proliferation of HCV29 treated with Y-vec CM or Y-shβ1 CM. **Figure S12.** Apoptosis of HCV29 treated with Y-vec CM or Y-shβ1 CM. **Figure S13.** Migratory ability of HCV29 treated with Y-vec CM or Y-shβ1 CM. **Figure S14.** Potential *N*-glycosylation sites (indicated by triangles) on integrin β1. Combined mutation to Asp of *N*-glycosylation sites 1–3, 4–6, 7–8 and 9–12 gave rise to Δ1-3, Δ4-6, Δ7-8 and Δ9-12. **Figure S15.** Sialic acid levels of MDA-MB-231 mutants, analyzed by lectin blotting. **Figure S16.** Migratory ability of HCV29 treated with CM from Y-vec, WT, Δ4–6 and Δ7–8 mutants. **Figure S17.** Proliferation of HCV29 treated with CM from Y-vec, WT, Δ4–6 and Δ7–8 mutants. **Figure S18.** Apoptosis of HCV29 treated with CM from Y-vec, WT, Δ4–6 and Δ7–8 mutants. **Table S1.** Information of plasma samples from bladder cancer patients and healthy subjects in Fig. 1I, J. **Table S2.** Information of plasma samples from bladder cancer patients and healthy subjects in Figs. 1M, N, 3N–P.

## Data Availability

The proteomic data have been deposited to the ProteomeXchange Consortium via the PRIDE, partner repository with the PXD036973 and 10.6019/PXD036973.

## Abbreviations

ASGPR: Asialoglycoprotein receptor
BSA: Bovine serum albumin
BCA: Bicinchoninic acid
CM: Conditioned medium
co-IP: Co-immunoprecipitation
DTT: Dithiothreitol
ECM: Extracellular matrix
ECL: Enhanced chemiluminescence
ELISA: Enzyme-linked immunosorbent assay
FBS: Fetal bovine serum
FN: Fibronectin
HCV29: Normal bladder epithelia
H&E: Haematoxylin and eosin
IP: Immunoprecipitation
MAL-II: *Maackia amurensis* Lectin, recognizing α2,3-linked sialic acids
MS: Mass spectrometry
NEU1: Sialidase-1 (neuraminidase 1)
Neu5Ac: *N*-Acetylneuraminic acid
Neu5Gc: *N*-Glycolylneuraminic acid
PBS: Phosphate buffered saline
PBST: Phosphate-buffered saline + Tween-20
RGD: Arg-Gly-Asp
sEV: Small extracellular vesicles
shRNA: Short hairpin RNA
SNA: *Sambucus nigra* lectin, recognizing α2,6-linked sialic acids
T24: Transitional cancers of human urine bladder
TBST: Tris-buffered saline + Tween-20
TMB: 3,3′,5,5′-Tetramethylbenzidine
YTS-1: Highly malignant invasive bladder cancer
