# Supplementary material for: Sialylation on vesicular integrin β1 determined endocytic entry of small extracellular vesicles into recipient cells
Source: Cell Mol Biol Lett. 2024 Apr 1;29:46. doi: 10.1186/s11658-024-00562-0 (PMC10983696; doi:10.1186/s11658-024-00562-0)
Supplement: Supplementary file 1 — Additional file 1: Figure S1. Proliferation of HCV29 treated with KK47 CM or YTS-1 CM. Figure S2. Apoptosis of HCV29 treated with KK47 CM or YTS-1 CM. Figure S3. Migratory ability of HCV29 treated with KK47 CM or YTS-1 CM. Figure S4. Knockdown of Rab27A in YTS-1. Figure S5. Migratory ability of HCV29 treated with Y-vec CM or Y-shRab27a CM. Figure S6. Proliferation of HCV29 treated with Y-vec CM or Y-shRab27a CM. Figure S7. Apoptosis of HCV29 treated with Y-vec CM or Y-shRab27a CM. Figure S8. Confocal microscopic imaging of sEV endocytosis. HCV29 were treated with fluorescence labeled YTS-1sEV. Figure S9. Levels of sialic acids, integrin β1, CD63 and TSG101 in sEV from integrin β1 silenced cells. Figure S10. shRNAs in sEV from Y-shβ1 cells by realtime PCR analysis. Figure S11. Proliferation of HCV29 treated with Y-vec CM or Y-shβ1 CM. Figure S12. Apoptosis of HCV29 treated with Y-vec CM or Y-shβ1 CM. Figure S13. Migratory ability of HCV29 treated with Y-vec CM or Y-shβ1 CM. Figure S14. Potential N-glycosylation sites (indicated by triangles) on integrin β1. Combined mutation to Asp of N-glycosylation sites 1–3, 4–6, 7–8 and 9–12 gave rise to Δ1-3, Δ4-6, Δ7-8 and Δ9-12. Figure S15. Sialic acid levels of MDA-MB-231 mutants, analyzed by lectin blotting. Figure S16. Migratory ability of HCV29 treated with CM from Y-vec, WT, Δ4–6 and Δ7–8 mutants. Figure S17. Proliferation of HCV29 treated with CM from Y-vec, WT, Δ4–6 and Δ7–8 mutants. Figure S18. Apoptosis of HCV29 treated with CM from Y-vec, WT, Δ4–6 and Δ7–8 mutants. Table S1. Information of plasma samples from bladder cancer patients and healthy subjects in Fig. 1I, J. Table S2. Information of plasma samples from bladder cancer patients and healthy subjects in Figs. 1M, N, 3N–P. [file 11658_2024_562_MOESM1_ESM.docx]

**Additional Information**

**Sialylation on vesicular integrin β1 determined endocytic entry of small extracellular vesicles into recipient cells**

Meixuan Lin^1^, Xiaoqiang Xu^1^, Xiaoman Zhou^1^, Hui Feng^1^, Ruili Wang^1^, Yunyun Yang^1^, Jing Li^1^, Ning Fan^1^, Yazhuo Jiang^2^, Xiang Li ^3^, Feng Guan^1,*^,

Zengqi Tan ^3,*^

^1^ Key Laboratory of Resource Biology and Biotechnology in Western China, Ministry of Education, Provincial Key Laboratory of Biotechnology, College of Life Sciences, Northwest University, Xi'an, China

^2^ Department of Urology, Provincial People’s Hospital, Xi’an, China

^3^ Institute of Hematology, School of Medicine, Northwest University, Xi'an, China

***Corresponding authors:** Zengqi Tan (zengqtan@nwu.edu.cn) School of Medicine, Northwest University, Xi'an, China, or Feng Guan (guanfeng@nwu.edu.cn) College of Life Sciences, Northwest University, Xi’an, China.


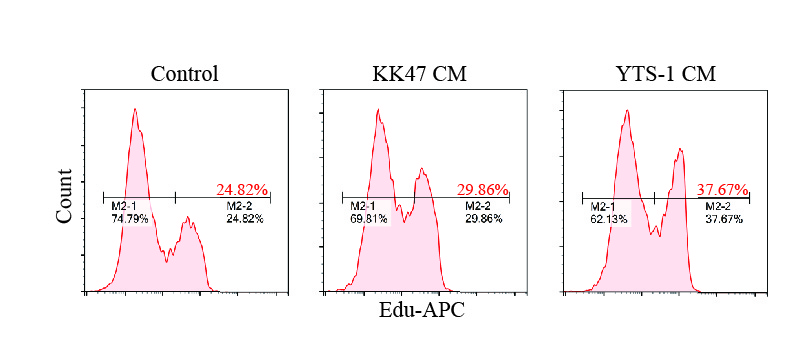


**Figure S1.** Proliferation of HCV29 treated with KK47 CM or YTS-1 CM.


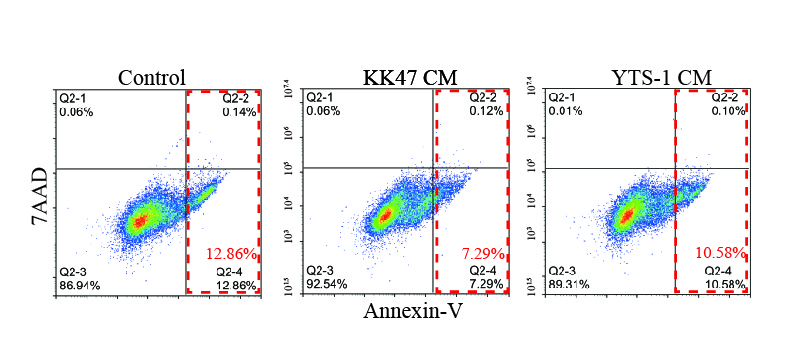


**Figure S2.** Apoptosis of HCV29 treated with KK47 CM or YTS-1 CM.


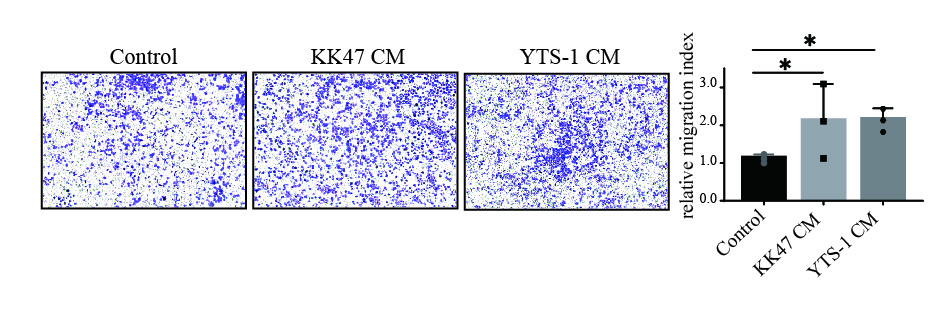


**Figure S3.** Migratory ability of HCV29 treated with KK47 CM or YTS-1 CM.


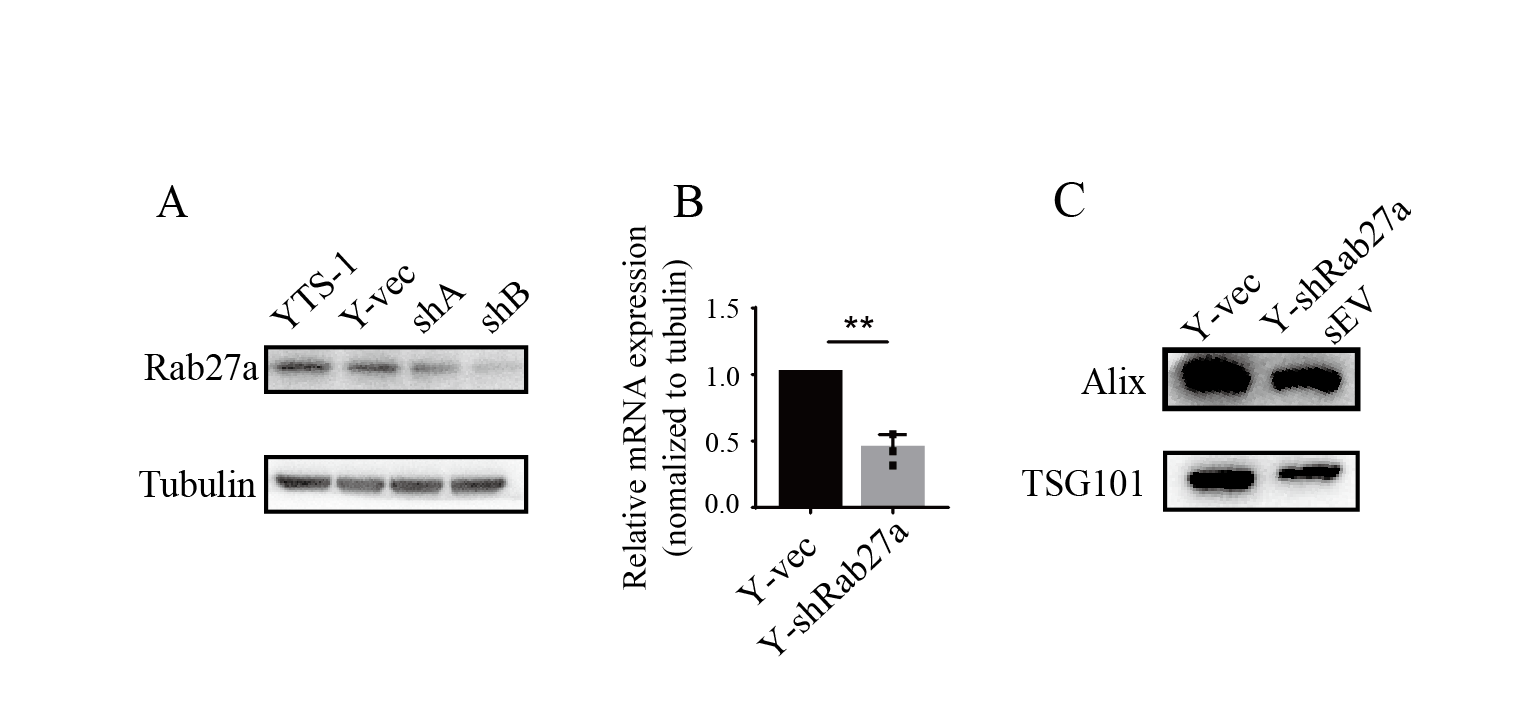


**Figure S4.** Knockdown of Rab27A in YTS-1. **(A)** Rab27a expression in Rab27a silenced YTS-1 cells (termed shA/B), analyzed by Western blotting. shB (termed Y-shRab27a) was used for further assay. **(B)** Relative mRNA expression of Rab27a in Y-shRab27a and Y-vec cells, analyzed by qPCR. **(C)** The expression of Alix and TSG101 in sEV from equal numbers of Y-shRab27a and Y-vec cells was analyzed by Western blotting.


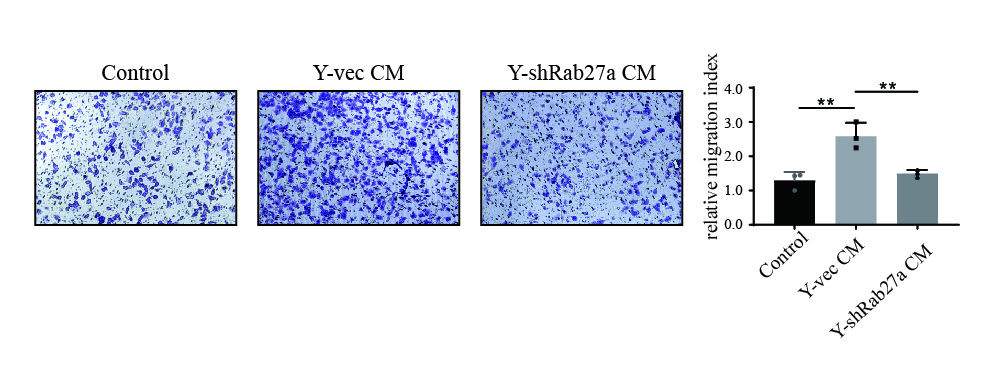


**Figure S5.** Migratory ability of HCV29 treated with Y-vec CM or Y-shRab27a CM.


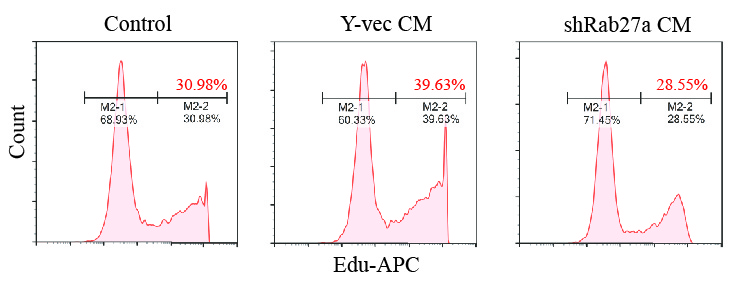


**Figure S6.** Proliferation of HCV29 treated with Y-vec CM or Y-shRab27a CM.


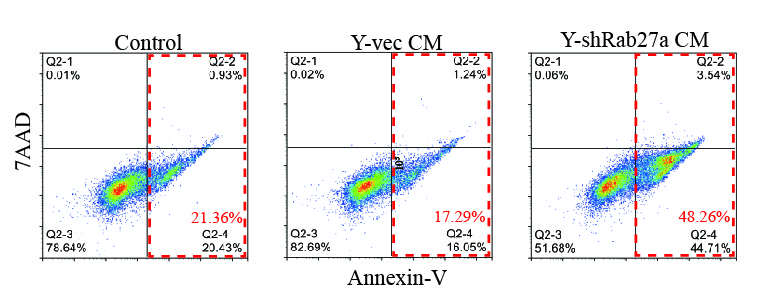


**Figure S7.** Apoptosis of HCV29 treated with Y-vec CM or Y-shRab27a CM.


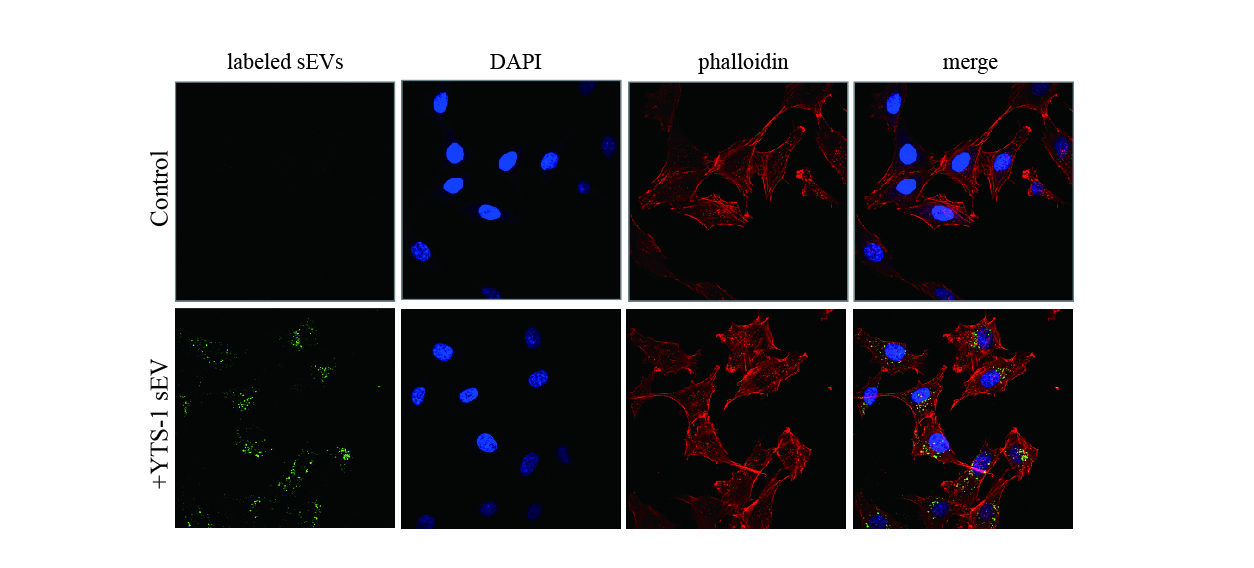


**Figure S8.** Confocal microscopic imaging of sEV endocytosis. HCV29 were treated with fluorescence labeled YTS-1sEV.


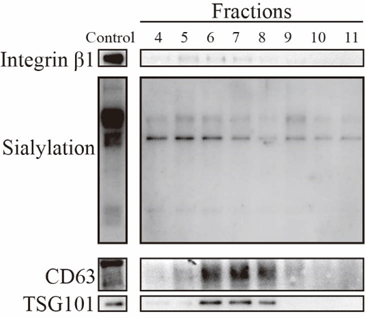


**Figure S9.** Levels of sialic acids, integrin β1, CD63 and TSG101 in sEV from integrin β1 silenced cells. Y-shβ1 sEV were purified by density gradient centrifugation. sEV and adjacent fractions (fractions 4-11) were collected for another ultracentrifugation and subjected to western blot. Levels of two sEV markers (TSG101, CD63) and integrin β1 were analyzed by western blotting, and sialic acid levels were analyzed by lectin blotting. Control: Y-vec sEV.


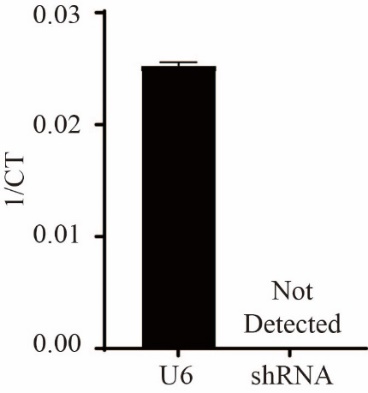


**Figure S10.** shRNAs in sEV from Y-shβ1 cells by realtime PCR analysis.


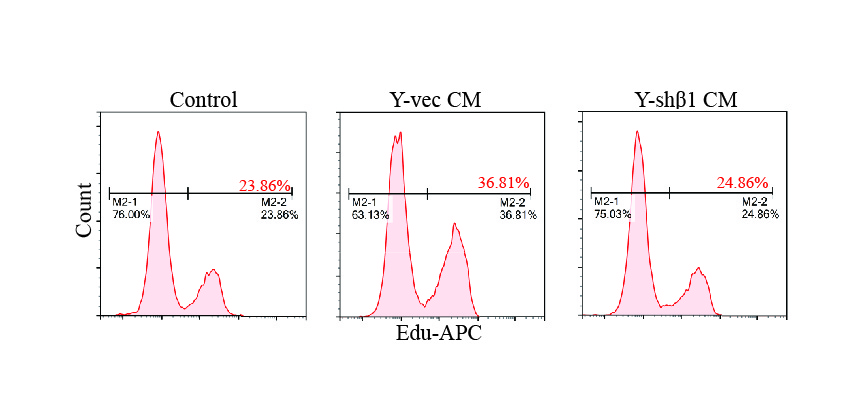


**Figure S11.** Proliferation of HCV29 treated with Y-vec CM or Y-shβ1 CM.


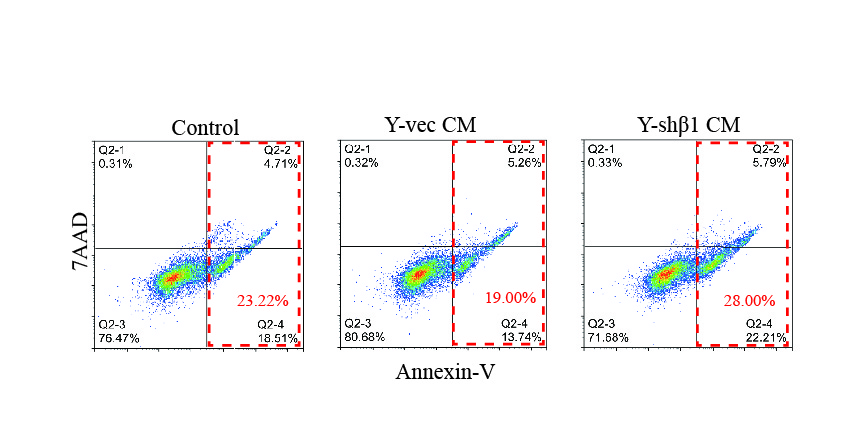


**Figure S12.** Apoptosis of HCV29 treated with Y-vec CM or Y-shβ1 CM.


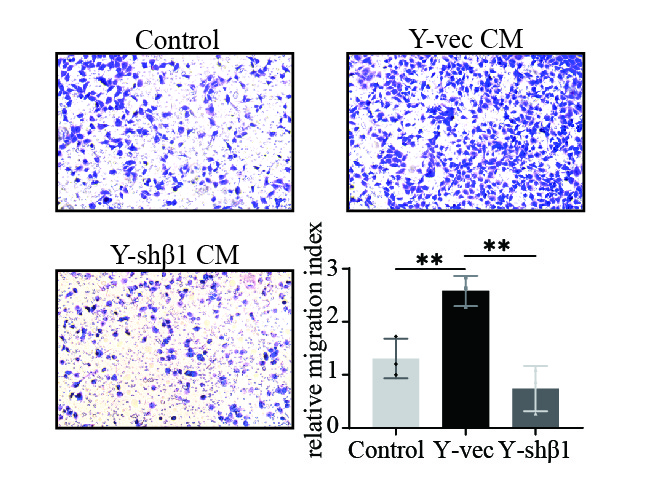


**Figure S13.** Migratory ability of HCV29 treated with Y-vec CM or Y-shβ1 CM.


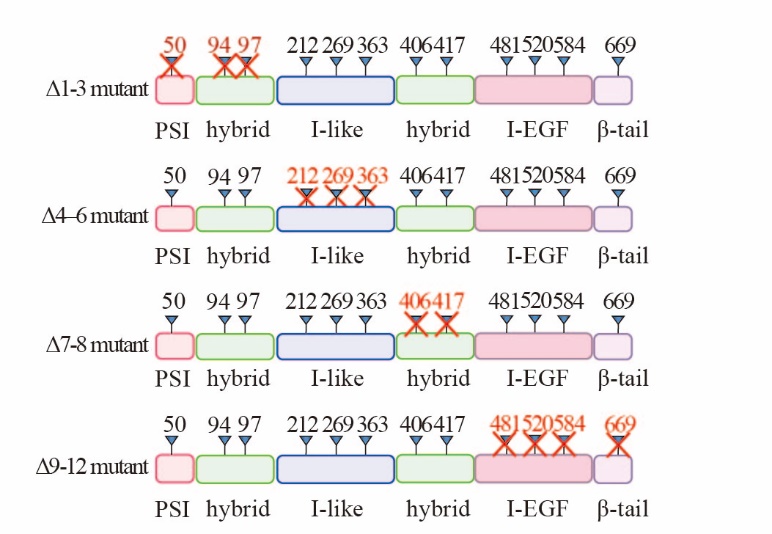


**Figure S14.** Potential N-glycosylation sites (indicated by triangles) on integrin β1. Combined mutation to Asp of N-glycosylation sites 1-3, 4-6, 7-8 and 9-12 gave rise to Δ1-3, Δ4-6, Δ7-8 and Δ9-12.


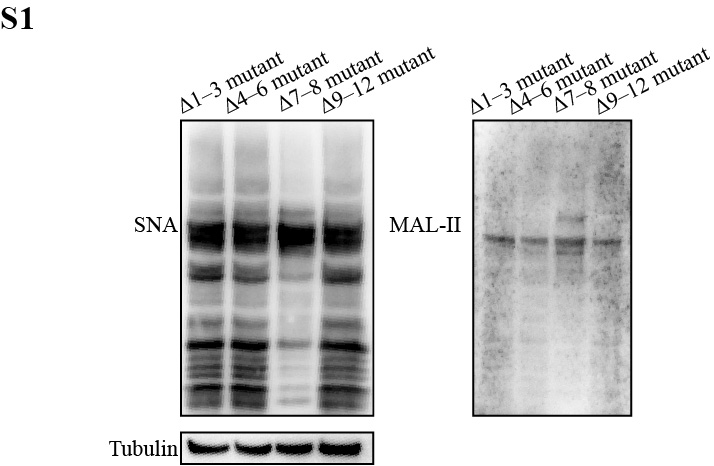


**Figure S15.** Sialic acid levels of MDA-MB-231 mutants, analyzed by lectin blotting.


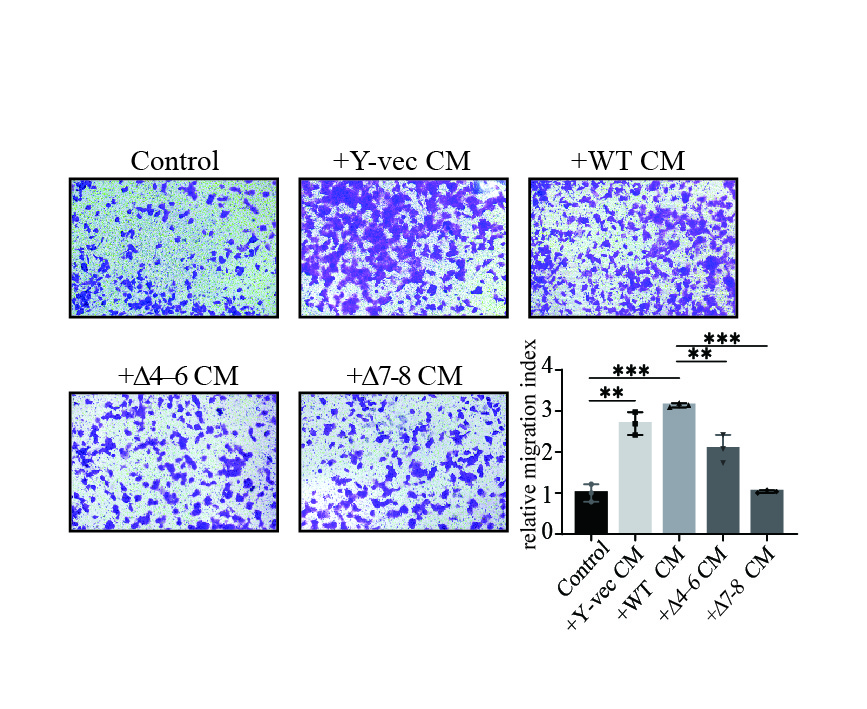


**Figure S16.** Migratory ability of HCV29 treated with CM from Y-vec, WT, Δ4–6 and Δ7–8 mutants.


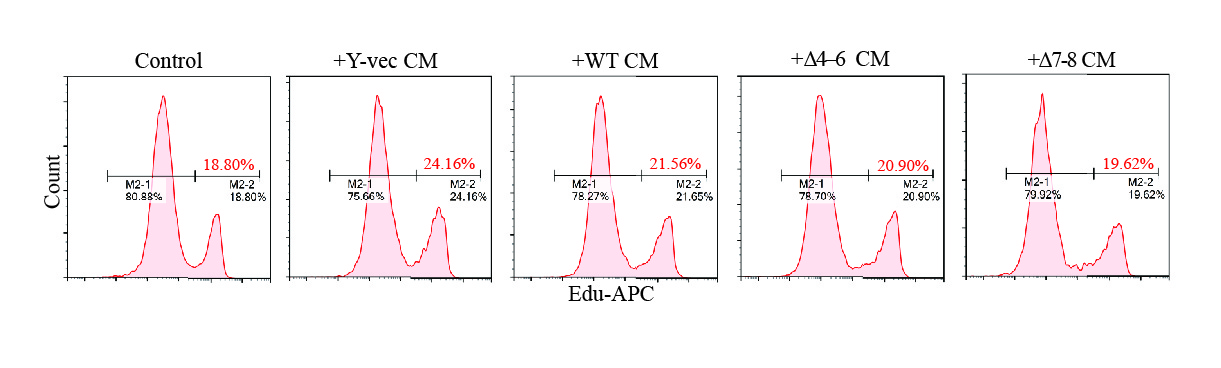


**Figure S17.** Proliferation of HCV29 treated with CM from Y-vec, WT, Δ4–6 and Δ7–8 mutants.


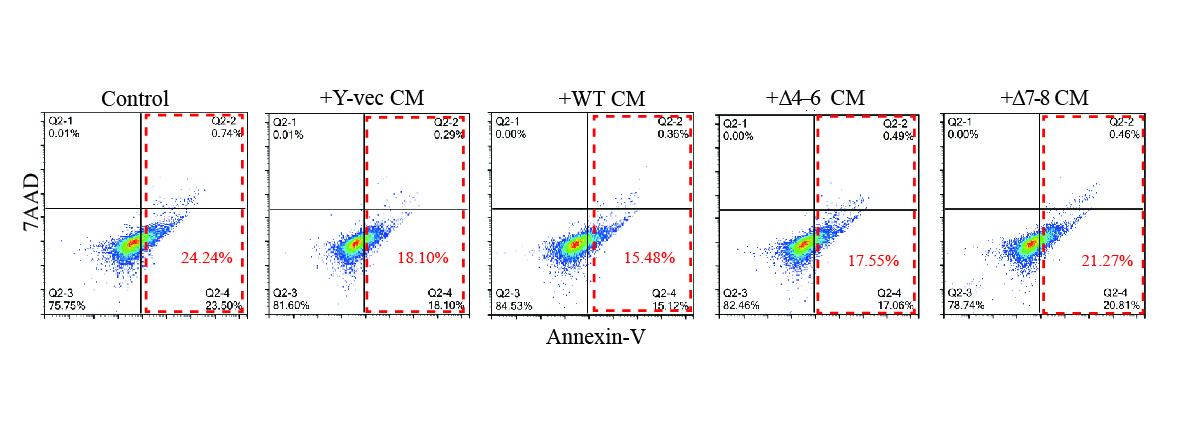


**Figure S18.** Apoptosis of HCV29 treated with CM from Y-vec, WT, Δ4–6 and Δ7–8 mutants.

**Table S1**. Information of plasma samples from bladder cancer patients and healthy subjects in Fig. 1I&J.

| Characteristics | healthy subjects (n=30) | bladder cancer patients (n=48) |
| --- | --- | --- |
| Age（Mean±SD） | 37±10 | 68±12 |
| Male | 15 | 42 |
| Female | 15 | 6 |
| Invasive cancer | - | 33 |
| Radiotherapy and/or chemotherapy | - | 19 |
| Tumor grading T1^#^ | - | 21 |
| Tumor grading T2 | - | 10 |
| Tumor grading T3 | - | 3 |
| Tumor grading T4 | - | 1 |

^#^ Some tumor grading information for certain patients is missing.

**Table S2**. Information of plasma samples from bladder cancer patients and healthy subjects in Fig. 1M, 1N, 3N, 3O, 3P.

| Characteristics | healthy subjects (n=17) | bladder cancer patients (n=48) |
| --- | --- | --- |
| Age（Mean±SD） | 50±15 | 68±12 |
| Male | 5 | 42 |
| Female | 12 | 6 |
| Invasive cancer | - | 33 |
| Radiotherapy and/or chemotherapy | - | 19 |
| Tumor grading T1^#^ | - | 21 |
| Tumor grading T2 | - | 10 |
| Tumor grading T3 | - | 3 |
| Tumor grading T4 | - | 1 |

^#^ Some tumor grading information for certain patients is missing.
